# Identification of Loci and Candidate Genes Analyses for Tocopherol Concentration of Soybean Seed

**DOI:** 10.3389/fpls.2020.539460

**Published:** 2020-09-09

**Authors:** Meinan Sui, Yan Jing, Haiyan Li, Yuhang Zhan, Jian Luo, Weili Teng, Lijuan Qiu, Hongkun Zheng, Wenbin Li, Xue Zhao, Yingpeng Han

**Affiliations:** ^1^ Key Laboratory of Soybean Biology in Chinese Ministry of Education (Key Laboratory of Soybean Biology and Breeding/Genetics of Chinese Agriculture Ministry), Northeast Agricultural University, Harbin, China; ^2^ Institute of Crop Science, National Key Facility for Crop Gene Resources and Genetic Improvement (NFCRI) Chinese Academy of Agricultural Sciences, Beijing, China; ^3^ Bioinformatics Division, Biomarker Technologies Corporation, Beijing, China

**Keywords:** soybean seed, tocopherol concentration, genome-wide association analysis, linkage mapping, candidate genes

## Abstract

Tocopherol (Toc) occurs in soybean seeds and is extracted together with the soybean oil. Toc is utilized as an antioxidant in food and an additive in animal feed. A total of 180 representative accessions and 144 recombinant inbred lines (RILs) from the cross of ‘Hefeng 25’ and ‘OAC Bayfield’ were selected to evaluate individuals and total Toc concentrations in soybean seeds. The 180 soybean samples were sequenced by the approach of Specific Locus Amplified Fragment Sequencing (SLAF-seq). A total of 22,611 single nucleotide polymorphisms (SNPs) were developed. Nineteen quantitative trait nucleotides (QTNs) were identified associated with individual or total-Toc based on genome-wide association analysis (GWAS). Among them, three QTNs located near known QTLs, and 16 were novel. Eighteen QTLs and nine eQTLs were also detected by linkage mapping. The QTN rs9337368 on Chr.02 was colocalized according to the linkage mapping of the RILs and genome-wide association analysis and regarded as a stable locus for mining the candidate genes in association with Toc. A total of 42 candidate genes near the 200 kbp flanking region of this identified locus were found. Upon a gene-based association, 11 SNPs from five genes out of the 42 candidates were detected. Expression level analysis of five candidate genes revealed that two genes were significantly related to Toc content. The identified loci, along with the candidate genes, might be valuable for increasing the Toc concentration in soybean seeds and improving the nutritional value of soybean oil.

## Introduction

Soybean (*Glycine max* (L.) Merr.) is a major crop for oil and food. Soybean oil accounts for 30% of vegetable oil consumption. Tocopherols (Tocs) are extracted with soybean oil during seed processing. Toc concentrations account only 1.5% of the total oil, but they are important for providing oxidative stability (Tavva et al., 2007). Toc is also used as an antioxidant in foods and a nutrient additive in animal food to improve meat quality (Munné-Bosch and Alegre, 2002). As a human diet supplement, Toc can reduce cancer and help prevent cardiovascular diseases (Dwiyanti et al., 2007; Shaw et al., 2017). Toc is a member of the family of structurally-related compounds, including *α*-, *β*-, *γ*- and *δ*-Toc, and four corresponding unsaturated derivatives, *α*-, *β*-, *γ*- and *δ*-tocotrienols (Wan et al., 2008). All Tocs are amphipathic molecules and contain a polar chromanol head group, which is derived from homogentisic acid and a lipophilic tail from phytyl-diphosphate (Maheswari et al., 2015; Park et al., 2019). In soybean oil, the biological activities of four individual Tocs vary with the number and position of methyl groups on the chromanol ring (Bramley et al., 2000). The biological activities of *α*-, *β*-, *γ*-, and *δ*-Toc are 100, 50, 10 and 3% equivalent to that of *α*-Toc activity (Kamal-Eldin and Appelqvist, 1996; Wan et al., 2008). The proportion of *α*-Toc is usually less than 10% of the total Toc in soybean seeds (Park et al., 2019). The demand for Toc has increased due to the increasing interest in functional foods and meat products. However, about 85% of Toc is derived from chemical synthesis nowadays (Dwiyanti et al., 2007). The biological activities of Toc that were synthesized by chemical account for only 50% of the natural *α*-Toc (Dwiyanti et al., 2007). Thus, increasing *α*-Toc proportion and total Toc concentration of soybean seeds is important for soybean breeding programs.

Toc content of soybean seeds is a complex quantitative trait that is regulated by multiple genetic and environmental factors (Seguin et al., 2010). Genetic improvement on Toc content of soybean seeds by traditional selection is inefficient and time-consuming. Marker-assisted selection (MAS) is faster and more accurate and is based on genotype instead of solely on phenotype. Determining the genetic basis of Toc in soybean seeds is critical for MAS. Two strategies, including traditional quantitative trait locus (QTL) mapping and genome-wide association study (GWAS), were utilized to determine the genetic basis of Toc. For bi-parent QTL mapping, only a limited number with famous strains that have genetic basis (‘Keszthelyi A.S,’ ‘OAC Bayfield’ and ‘Beifeng 9’) have been analyzed (Li et al., 2010; Shaw et al., 2016). Nearly all of these identified QTLs were obtained through a low-resolution genetic map, which was constructed by lower density simple-sequence repeat (SSR) markers (Li et al., 2010; Shaw et al., 2016). These identified QTLs were difficult to use for MAS. Complementary to linkage analysis, genome-wide association study (GWAS) with a population of unrelated lines and high-density single nucleotide polymorphism (SNP) markers could identify causal genes for a broad range of complex traits. Many important traits including soybean cyst nematode (Han et al., 2015; Zhang et al., 2016), seed weight (Liu et al., 2017), fatty acid content (Zhao et al., 2019), protein and oil (Li et al., 2018; Lee et al., 2019) have been reported in the Soybase database. However, no studies for GWAS of Toc concentration in soybean seeds have been reported.

The Toc biosynthetic pathway to higher plants is known, and relatively few genes methyl-6-phytyl-1, 4-benzoquinone (*MPBQ*), tocopherol cyclize (*TC*) and Phytyl transferase (*HPT*)) are associated with Toc accumulation. Increasing the transcript abundance of these genes is an effective approach for improving Toc concentration (Dwiyanti et al., 2007). The integrated analysis of genotype and transcript abundance data associated with complex traits can be used to identify novel genetic pathways involved in complex traits through QTL expression (Wang et al., 2014). Wang et al. (2014) identified 33 eQTLs of four genes from the biosynthetic pathway of soybean seeds isoflavone, and these were valuable for MAS.

In this study, a GWAS of Toc concentration was performed through 22,611 SNPs and 180 soybean accessions. Based on a recombinant inbred line (RIL) population, a QTL analysis was conducted, and *MPBQ*, *TC*, and *HPT* in the Toc biosynthetic pathway were selected as the target genes (TGs) to analyze eQTL that related to Toc concentration. This study aims to identify stable loci and screen candidate genes that highly associated with individual or total-Toc concentration.

## Materials and Methods

### Plant Material and Field Trials

A germplasm collection including 180 diverse soybean accessions, representing the genetic diversity inside and outside of China (**Table S1**), was selected to evaluate the individual and total Toc and used for subsequent sequencing analysis and GWAS. Additionally, a cross population of 144 recombinant inbred lines (RILs), derived from crossing between two elite soybean cultivars, ‘Hefeng 25’ (low Toc concentration) and ‘OAC Bayfield’ (high Toc concentration), was used for subsequent linkage analysis (Tanner et al., 1998; Guan et al., 2009). All samples were planted in Harbin for four consecutive years (2014–2017). Field trials were performed using single-row plots (3 m long and 0.65 m between rows) and a randomized complete block design (three replications per test environment). After reaching full maturity, ten randomly selected plants per row in each plot were collected and used to evaluate individual and total Toc concentration. Additionally, for the eQTL analysis, immature seeds of the RILs at the R7 reproductive stage were harvested and quantified in 2017 for transcript abundances (Li et al., 2016). Furthermore, sixteen characteristic germplasms within the association panel were used to analyze the expression levels of candidate genes, including relatively higher accessions (Line 1 to Line 8) and lower accessions (Line 9 to Line 16) of *α*-Toc, *γ*-Toc, *δ*-Toc, and total Toc contents were planted in 2018. Immature seeds were harvested at the R7 reproductive stage, which stage showed the highest content of Toc in grains, for the determination of Toc content and expression analysis of genes (Li et al., 2016).

### Sample Preparation and High-Performance Liquid Chromatography (HPLC) Analysis

Extraction and determination of soybean seed Toc were conducted based on methods described by Ujiie et al. (2005). In detail, 5 g test samples were ground to a fine powder. A mixture of 0.1 g soybean flour, 0.05 g ascorbic acid, and 3 ml of 80% (w/v) ethanol was stirred in a 5 ml tube for 10 s, sonicated for 15 min, and stirred for 10 min after adding 2 ml of hexyl hydride at room temperature. The slurry was centrifuged at 13,000 g for 15 min, and then the clear aliquot was filtered through a 0.45-µm PTFE filter. The supernatant was used to measure individual and total Toc using HPLC (Dionex ASI-100, USA) with a C18 reverse-phase column (250 mm length and 4.6 µm particle sizes). The measurement conditions (solvent A: 100% methanol, flow rate: 1.5 ml min^−1^, the temperature of the column: 40°C; and the injection volume: 20 μl) were set. A senex fluorescent light a detector was used with excitation at 295 nm and emission at 330 nm. The standard concentrations ranged from 5 to 100 µM, and 10 µl volumes (5–1,000 pM) were injected. FR spectra were recorded, and the responses were integrated using Dionex 2.0 software. The external standard method was used for the quantification of Toc in soybean seeds.

### Real-Time PCR of the Target Genes Involved in the Toc Biosynthetic Pathway

Some target genes (TGs) involved in the biosynthesis pathway of soybean tocopherols (https://www.kegg.jp/) were selected for investigation of transcript abundance. Total RNA was isolated from soybean seeds samples of all the 144 RIL individuals at the R7 stage using plant RNA purification reagent (TIANGEN, DP432), and then we synthesized the first-strand cDNA based on the TIANScript RT Kit (TIANGEN, KR104). The transcript abundances of the TGs were determined by real-time PCR analysis, and the assay was performed on an ABI 7500 Fast using SuperReal PreMix Plus (SYBR Green) Kit (TIANGEN, FP205) according to manufacturer instructions.

Each reverse transcription was performed with approximately 1 μg of total RNA. About 1 μl of the first-strand cDNA, 0.2 μM of each primer, 0.4 μl of DyeII, and 10 μl of SYBR Green PCR Master Mix, were used for each amplification reaction of 20 μl. The real-time PCR programs were as follows: 95°C for 30 s, for holding stage; 95°C for 3 s, 60°C for 30 s, 72°C for 30 s for 40 cycles, for cycling stage; 95°C for 15 s, 60°C for 1 min, 95°C for 30 s and 60°C for 15 s for melt curve stage. Three technical replicates and three biological duplications were conducted for each sample, and the relative transcript levels were calculated using the comparative threshold method (2^−△△CT^) with *GmActin4* (GenBank accession no. AF049106) as the internal standard control. The sequences of the primer pairs used to amplify these genes are listed in **Table S2**.

### SNP Genotyping Data Collection

The genomic DNA of the whole association panel was isolated using the CTAB method described by Han et al. (2015) and then sequenced based on the specific-locus amplified fragment sequencing (SLAF-seq) approach (Sun et al., 2013). A group of digest enzymes Mse I (EC 3.1.21.4) and HaeIII (EC:3.1.21.4) (Thermo Fisher Scientific Inc., Waltham, MA, USA.) were used to obtain more than 50,000 sequencing tags, each 300–500 bp in length, from each accession based on preliminary analysis of the reference genome. These tags were distributed in unique genomic regions of the 20 soybean chromosomes. The sequencing libraries of each accession were defined based on the sequencing tags. For each accession library, the 45 bp sequence reads at both ends of the sequencing tags were obtained through a barcode method combination with Illumina Genome Analyzer II system (Illumina Inc., San Diego, CA, USA). The alignment between the raw paired-end reads and soybean reference genome was performed using Short Oligonucleotide Alignment Program 2 (SOAP2) software. The raw read in the same genomic position were used to define the SLAF groups based on more than 58,000 high-quality SLAF tags from each tested sample. The SNPs were based on MAF ≥ 0.05. The genotype was regarded as heterozygous when the depth of the minor allele/the total depth of the sample was more than 1/3 (Han et al., 2016).

Twenty soybean germplasms (10 lines with higher level and 10 lines with lower level of Toc concentration) were selected for a genome re-sequencing 10-fold in depth using an Illumina HiSeq 2500 sequencer. Paired-end re-sequencing reads were mapped to the reference genome *via* BWA (Version: 0.6.1-r104) using default parameters. The BAM format of these mapped reads was converted *via* SAMtools48 (Version: 0.1.18) software, and unmapped and non-unique reads were filtered. Duplicated reads were further filtered with the Picard package (picard.sourceforge.net, Version:1.87). The BEDtools (Version: 2.17.0) coverage Bed program was used for computing the coverage of sequence alignments. A sequence was defined as “absent” when coverage was lower than 90% and “present” when coverage was higher than 90%. SNPs were identified through the Genome Analysis Toolkit (GATK, version 2.4-7-g5e89f01) and SAMtools (Zhou et al., 2015). Only the SNPs which were detected by both methods were further analyzed. SNPs with allele frequencies lower than 1% in the population were discarded. The annotations of SNPs were conducted based on the soybean reference genome using the package ANNOVAR (Version: 2013-08-23).

### Population Structure Evaluation and Linkage Disequilibrium (LD) Analysis

Principal component analysis (PCA) programs of Software package GAPIT (Lipka et al., 2012) were used to analyze the population structure of the natural panel. Software TASSEL version 3.0 (Bradbury et al., 2007) was used to determine LD across the soybean genome with these SNPs (MAF ≥ 0.05 and missing data < 3%). Compared with the GWAS, missing SNP genotypes were not imputed with the major allele before LD analysis. Parameters in the program included MAF (≥ 0.05) and the integrity of each SNP (> 80%).

### Genome-Wide Association Analysis

The association signals of individuals and total Toc were identified based on 22,611 SNPs and 180 tested soybean samples with the compressed mixed linear model (CMLM) in GAPIT (Lipka et al., 2012). The significance threshold for the association between SNP and traits was determined by −log_10_(P) ≥ 3 (Yan et al., 2017).

### QTL and eQTL Mapping Based on Linkage Analysis for Soybean Toc Concentration-Related

QTL and eQTL analyses were performed using the composite interval mapping (CIM) method implemented with QTL IciMapping v4.1 using stepwise regression for cofactor selection (Meng et al., 2015). The LOD score threshold was determined based on the results of 1,000 permutations for each trait. The percentage of phenotypic variance and additive effect explained by a QTL for a trait was also estimated.

### Prediction of Candidate Genes Controlling Toc Concentration of Soybean Seed

Candidate genes, located in the 200-kb genomic region up- and down-stream of each peak SNP, were classified and then annotated with the soybean reference genome through the methods developed by Cheng et al. (2017). The SNP variations that occurred in the regions of candidate genes, including exon regions, splicing sites, 5′UTRs and 3′UTRs, intron regions and upstream and downstream regions, were detected *via* genome re-sequencing data. A candidate gene-based association was conducted based on these SNPs and phenotype values of 20 soybean germplasms in different years by using the General Linear Model (GLM) model in TASSEL software 3.0 to identify haplotypes of candidate genes that related to Toc (Bradbury et al., 2007). Significant SNPs related to the target traits were claimed when the test statistic was *p* < 0.01. Permutation test was used to control the false positive results (Chaubey et al., 1993). The expression levels of five candidate genes in 16 characteristic soybean germplasms at R7 stage were detected by real-time PCR to further analyze candidate genes. The method of real-time PCR was the same as the expression analysis of target genes involved in the Toc biosynthetic pathway. The sequences of the primer pairs used to amplify these genes were listed in **Table S2**.

### Statistical Analysis

A statistical analysis was performed by SPSS 22.0. Descriptive statistics such as minimum, maximum, mean, coefficient of variation, skewness, and kurtosis were calculated for the selected population, including the tocopherol contents of 178 soybean germplasms in four environments, the tocopherol contents of RILs and the expression patterns of three genes (*MPBQ*, *TC*, *HPT*) in RILs at R7 stage. In addition, descriptive statistics such as minimum, maximum, mean, standard error, and coefficient of variation were calculated for expression levels of candidate genes in 16 characteristic soybean germplasms. Analysis of Pearson’s correlation coefficient among tocopherol contents related to expression levels of candidate genes were estimated. The degree of correlation was divided into two levels, including significant correlation (P < 0.05) and extremely significant correlation (P < 0.01).

## Results

### Phenotype Variation Analysis of the Germplasm Collection and the RILs

**Figure 1:**
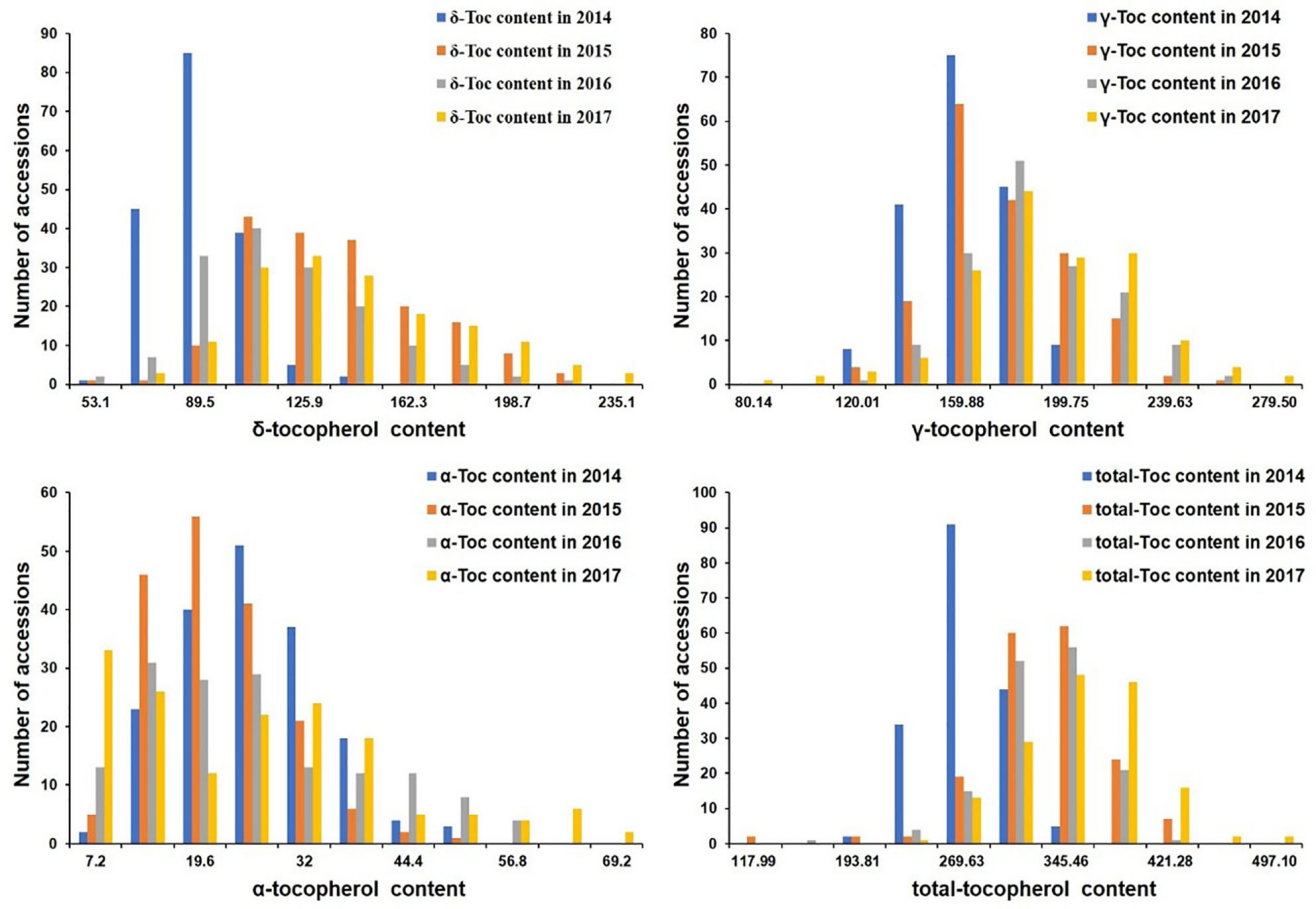
Distribution of tocopherol content among 180 soybean accessions in four tested environments.

The phenotypic values were measured to analyze the variation distribution of the association panel at Harbin from 2014 to 2017 and the RIL population at Harbin in 2017 (**Tables 1** and **2**). In the association panel, the individual and total Toc concentration of the 180 tested soybean lines varied greatly, including *δ*-Toc (from 34.90 to 235.10 ug/g), *γ*-Toc (from 60.20 to 279.40 ug/g), *α*-Toc (from 1.00 to 69.20 ug/g) and total-Toc (from 80.08 to 497.10 ug/g). The contents of 112.36, 168.04, 21.59, and 301.34 ug/g were the means of the four years of data (**Table 1**). Of the RIL population, these concentrations also varied over a wide range with averages of 68.95, 139.82, 9.90, and 222.43 ug/g (**Table 2**). The coefficient of variation, skewness, and kurtosis of the association panel and RIL population are shown in **Tables 1** and **2**. Continuous distributions with no significant skewness or kurtosis of individual and total Toc concentration for the association and RIL populations showed that these phenotypic values were appropriate for the subsequent GWAS and QTL analysis (**Table 1**, **Figure 1** and **Table 2**). Additionally, the transcript abundances of three TGs (*MPBQ*, *HPT*, and *TC*) among the RILs at the R7 stage were measured (**Table 1**). Among these, the variation range of *TC* expression of the RILs was greater than the *MPBQ* and *HPT* expressions. No significant skewness or kurtosis of the three expressions was observed among the RILs; thus these values were suitable for the eQTL analysis.

**Table 1:** Basic genetic parameter statistics for tocopherol in the tested soybean population (n =180).

Trait	Year	Location	Min (ug/g)	Max (ug/g)	Mean (ug/g)	CV (%)	Skewness	Kurtosis
*δ*-Toc	2014	Harbin	52.68	130.65	81.02	2.22	0.77	0.75
	2015	Harbin	43.48	215.10	127.27	9.57	0.45	−0.05
	2016	Harbin	34.90	217.70	108.88	8.96	0.69	0.92
	2017	Harbin	65.70	235.10	132.29	13.11	0.58	−0.04
*γ*-Toc	2014	Harbin	105.94	193.58	150.53	2.89	0.05	−0.18
	2015	Harbin	86.97	244.70	165.11	6.78	0.34	0.08
	2016	Harbin	83.00	241.40	176.16	7.24	0.04	0.25
	2017	Harbin	60.20	279.40	180.38	11.13	−0.11	0.95
*α*-Toc	2014	Harbin	6.15	48.94	22.77	0.70	0.39	0.03
	2015	Harbin	2.96	48.43	18.40	0.58	0.82	0.84
	2016	Harbin	1.00	54.40	22.53	1.68	0.55	−0.53
	2017	Harbin	1.00	69.20	22.65	2.64	0.73	−0.01
total-Toc	2014	Harbin	183.62	332.03	254.75	7.19	0.18	−0.09
	2015	Harbin	80.08	416.04	307.72	21.11	−1.23	4.99
	2016	Harbin	130.30	405.80	307.56	15.43	−0.87	2.25
	2017	Harbin	222.50	497.10	335.32	22.37	0.18	0.66

Min, minimum; Max, maximum; CV, coefficient of variation.

**Table 2:** Total and individual tocopherol content of the RIL population.

Traits	Min(ug/g)	Max(ug/g)	Mean(ug/g)	CV (%)	Skewness	Kurtosis
*δ*-Toc	10.78	122.70	68.95	30.32	0.03	−0.46
*γ*-Toc	59.81	195.97	139.82	19.57	−0.32	0.08
*α*-Toc	0.10	25.53	9.90	93.46	0.27	−0.92
total-Toc	66.34	309.56	222.43	18.08	−0.52	1.42
*MPBQ* expression	0.46	267.61	59.44	97.07	1.08	1.40
*HPT* expression	1.18	39.66	13.39	76.61	0.89	0.47
*TC* expression	10.06	3236.01	1047.16	76.23	1.04	1.14

Min, minimum; Max, maximum; CV, coefficient of variation.

### SNP Genotyping of the Association Panel

**Figure 2:**
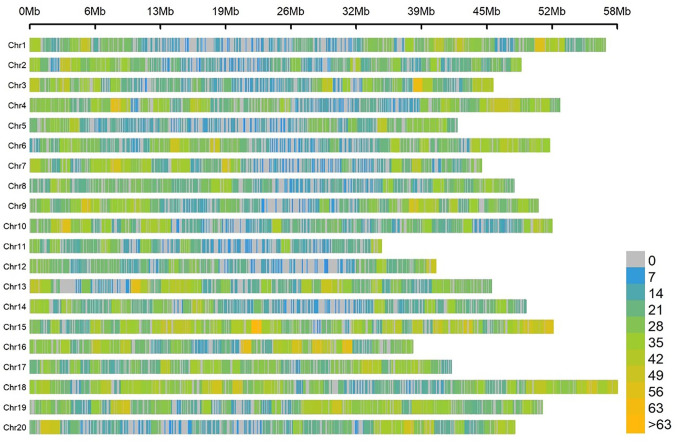
SNP density and distribution across the 20 soybean chromosomes.

For the association panel, the genomic DNA of the 180 soybean samples was sequenced based on the SLAF-seq approach. A total of 22,611 SNPs distributed on 20 soybean chromosomes were screened with MAF ≥ 0.05 and missing data < 3%. These SNPs spanned 947.07 Mb, which covered about 86.10% of the entire soybean genome (**Figure 2**). The number of SNPs varied across the 20 soybean chromosomes, and the average number of SNPs per chromosome was 1130. The average marker density was approximately one SNP every 41.89 kb genome-wide.

### Polymorphic SSRs for the RIL Population

**Figure 3:**
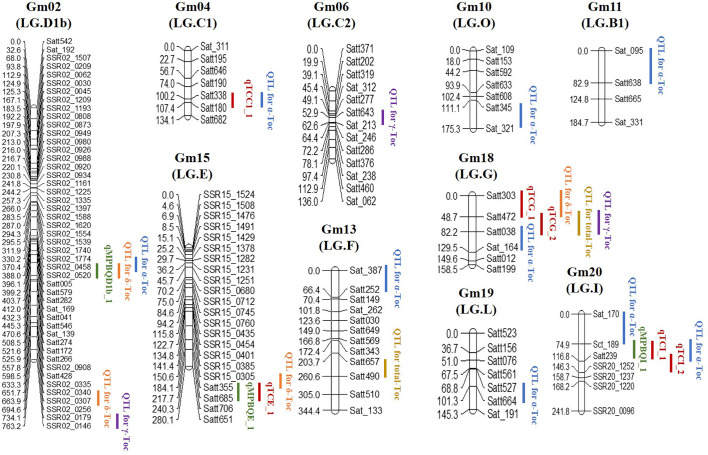
Summary of eQTL and QTL locations detected in the soybean genome.

Underlying the SSR markers mapped on the 20 LGs of soybean (Cregan et al., 1999; Song et al., 2004), 69 polymorphic SSRs between the two parental genotypes in this study were the new markers compared to the ones on the genetic map published by Li et al. (2010) and were applied in screening the RIL population. The genotypes of the recombinants were determined on the basis of these polymorphic markers and used to encrypt the reported genetic map for further QTL and eQTL analysis (**Figure 3** and **Table S3**).

### Genome-Wide Association of Toc Concentration in Soybean Seeds

**Figure 4:**
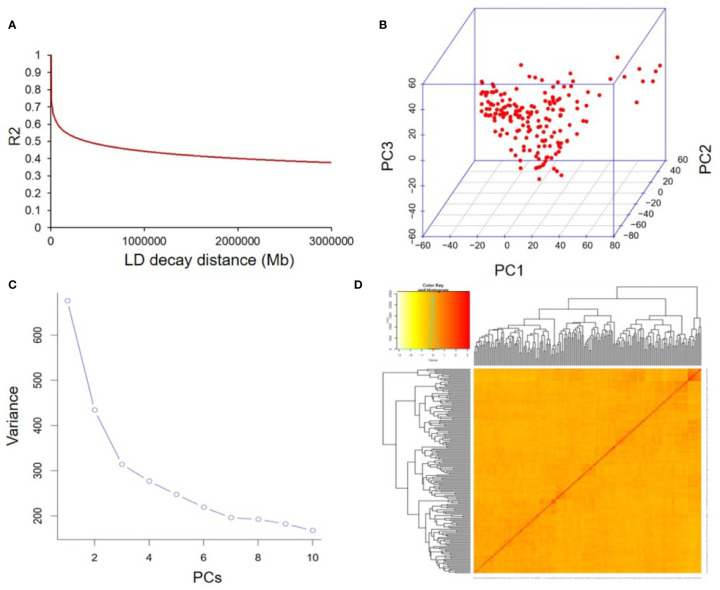
The linkage disequilibrium (LD), principal component, and kinship analyses of soybean genetic data. **(A)** The linkage disequilibrium (LD) decay of genome-wide association study (GWAS) population. **(B)** The first three principal components of the more than 20,000 SNPs used in the GWAS. **(C)** Population structure of soybean germplasm collection as reflected by principal components. **(D)** A heatmap of the kinship matrix of the 180 soybean accessions calculated from the same SNPs.

The average distance of LD decay was analyzed to describe the mapping resolution of GWAS. The mean LD decay of the panel was estimated as 223.2 kb when R^2^ dropped to 0.4 (**Figure 4A**). Principal component (PC) and kinship analyses were also conducted by the 22,611 SNPs. The first three PCs accounted for 12.83% of the overall genetic variation, and the inflection point occurred at PC3, which indicated that the first three PCs could dominate the population structure for the association mapping (**Figures 4B, C**). In addition, a lower level of genetic relatedness was found based on the distribution of the pairwise relative kinship coefficients among the 180 tested accessions (**Figure 4D**). Based on the CMLM model (Lipka et al., 2012), 19 SNP loci identified in at least two years were considered to be associated with individual or total Toc of soybean seeds. They distributed on ten different chromosomes (Chr.), including Chr.01, Chr.02, Chr.06, Chr.07, Chr.09, Chr.11, Chr.15, Chr.17, Chr.18, and Chr.20 (**Figure S1** and **Table 3**). Among them, three QTNs located near the SNP markers of rs37703714 on Chr.17, rs33069111, and rs35562231 on Chr.20, were near the genomic region of known QTLs for Toc concentration. The other 16 QTNs were novel Toc-related loci (**Table 3**). Of these QTNs, 15 loci located on different genomic regions of nine chromosomes were identified to be associated with total Toc of soybean seeds. The numbers of the QTNs correlated with *α*-Toc, *γ*-Toc and *δ*-Toc were 2, 5, and 12, respectively. The allele effects of these identified QTNs showed that different alleles for each QTN could significantly affect the Toc content of the tested soybean samples (**Table 3**).

**Table 3:** All significant SNPs for tocopherol traits detected by GWAS.

SNP	Chr.	Position	Year	Trait	-log10(P)	MAF	R^2^	KnownQTL		Allele1/Allele2	Average tocopherol of accessions with Allele1/Allele2 (ug/g)	Average tocopherol of population(ug/g)
rs2162876	1	2162876	2015	*α*-tocopherol	4.35	0.06	0.24			C/A	23.25/18.11	18.40
			2017	*α*-tocopherol	3.43	0.07	0.40				37.41/23.14	24.17
rs5437879	1	5437879	2015	total-tocopherol	3.17	0.06	0.13			A/C	343.91/305.56	307.72
			2015	*α*-tocopherol	4.36	0.06	0.24				25.91/17.95	18.40
rs5957934	1	5957934	2014	*δ*-tocopherol	4.05	0.41	0.32			G/A	86.95/76.85	81.02
			2016	*δ*-tocopherol	3.04	0.39	0.19				120.20/101.54	108.88
rs23631390	1	23631390	2015	total-tocopherol	3.85	0.05	0.14			G/A	311.36/203.01	307.72
			2015	*γ*-tocopheral	5.26	0.05	0.25				166.20/156.08	165.11
rs56799896	1	56799896	2017	total-tocopherol	3.08	0.06	0.22			T/G	337.83/336.20	336.08
			2017	*δ*-tocopherol	3.83	0.06	0.24				134.04/131.06	130.15
rs9337368	2	9337368	2015	total-tocopherol	3.46	0.06	0.13			A/T	311.06/226.82	307.72
			2015	*δ*-tocopherol	4.59	0.06	0.29				128.84/88.04	127.27
rs11882008	6	11882008	2014	total-tocopherol	3.02	0.08	0.26			A/C	272.69/253.07	254.75
			2014	*γ*-tocopheral	3.02	0.08	0.33				160.84/149.58	150.53
rs36073631	7	36073631	2017	total-tocopherol	4.08	0.04	0.25			C/G	350.50/335.09	336.08
			2017	*δ*-tocopherol	3.20	0.04	0.22				136.16/129.73	130.15
rs5307458	9	5307458	2017	total-tocopherol	3.72	0.19	0.24			T/G	339.28/335.61	336.08
			2017	*δ*-tocopherol	4.35	0.19	0.25				122.56/132.39	130.15
rs5553040	9	5553040	2014	*δ*-tocopherol	3.58	0.06	0.31			A/G	81.90/65.39	81.02
			2016	*δ*-tocopherol	3.13	0.06	0.19				111.09/73.87	108.88
rs20628671	11	20628671	2017	total-tocopherol	3.40	0.05	0.23			T/G	359.43/334.12	336.08
			2017	*δ*-tocopherol	4.29	0.05	0.25				137.23/130.24	130.15
rs30665207	15	30665207	2016	total-tocopherol	4.23	0.07	0.15			C/A	311.22/255.44	307.56
			2016	*γ*-tocopheral	3.05	0.07	0.26				178.01/148.12	176.16
rs14108110	17	14108110	2015	total-tocopherol	5.27	0.19	0.18			T/C	316.41/270.88	307.72
			2015	*δ*-tocopherol	3.45	0.19	0.26				130.29/113.97	127.27
rs14593163	17	14593163	2015	total-tocopherol	5.18	0.19	0.18			T/G	316.53/271.50	307.72
			2015	*δ*-tocopherol	3.49	0.19	0.26				130.19/115.46	127.27
rs14680873	17	14680873	2015	total-tocopherol	5.02	0.19	0.17			A/G	316.27/271.50	307.72
			2015	*δ*-tocopherol	3.29	0.19	0.26				129.96/115.46	127.27
rs37703714	17	37703714	2016	total-tocopherol	3.16	0.09	0.12	Seed tocopherol, gamma 3-6		G/A	310.99/267.81	307.56
			2016	*γ*-tocopheral	3.23	0.09	0.26				178.49/156.27	176.16
rs14143396	18	14143396	2014	*δ*-tocopherol	3.26	0.18	0.31			T/G	87.53/79.35	81.02
			2017	*δ*-tocopherol	3.38	0.19	0.23				146.46/125.66	130.15
rs33069111	20	33069111	2014	total-tocopherol	4.29	0.06	0.29	Seed tocopherol, total 3-3		A/G	290.83/252.11	254.75
			2014	*δ*-tocopherol	3.63	0.06	0.31				98.16/79.80	81.02
rs35562231	20	35562231	2015	total-tocopherol	3.51	0.23	0.14	Seed tocopherol, total 3-3		A/T	311.74/293.70	307.72
			2015	*γ*-tocopheral	3.82	0.23	0.21				169.48/150.07	165.11

### Linkage Mapping for Soybean Seeds Toc Concentration

For the linkage analysis, 18 QTLs (*α*-Toc, nine QTLs; *δ*-Toc, four QTLs; *γ*-Toc, three QTLs; total Toc, two QTLs) mapped on ten different chromosomes were detected using the CIM method (Meng et al., 2015). The phenotypic variation (PVE) explained by these QTLs was analyzed with the value range of 0.60–17.97% (**Table 4**). Additionally, additive effects analysis for individual or total Toc content was also performed. As shown in **Table 4**, two QTLs located in the Satt303–Satt472 and Satt472–Satt038 interval on Chr.18 for *δ*-Toc and total-Toc showed a higher positive additive effect. In contrast, two QTLs located in the interval of SSR02_0458–SSR02_0520 on Chr.02 and Satt657–Satt490 on Chr.13 exhibited a higher negative additive effect for *δ*-Toc and total-Toc, respectively. To confirm the stable loci for the Toc concentration, an eQTL analysis was conducted based on the same SSR markers and the transcript abundance of the TGs (**Table 5**). Finally, nine eQTLs mapped on five different chromosomes (Chr.02, Chr.04, Chr.15, Chr.18, and Chr.20) were detected. Among these, qMPBQD1b_1 on Chr.02, qMPBQE_1 on Chr.15, and qMPBQI_1 on Chr.20 were associated with *MPBQ* transcript abundance and could account for 2.07, 6.53, and 7.15% of the phenotypic variation, respectively. The other six eQTLs (qTCC1_1, qTCE_1, qTCG_1, qTCG_2, qTCI_1, and qTCI_2) underlying *TC* transcript abundance could explain the phenotypic variations from 2.86 to 7.20%. Of them, qTCG_2 located in the interval of Satt472–Satt038 on Chr.18 showed a higher LOD score with the value of 6.18.

**Table 4:** QTL information mapped in RIL population.

Trait	Gm (LG)	Position	Marker interval	Physical location ofMarkers	LOD	Phenotypic variationEffect (%)	Additive effect
*δ*-Toc	2(D1b)	386	SSR02_0458– SSR02_0520	8328227–9943059	5.2282	9.8437	−13.718
*δ*-Toc	2(D1b)	652	SSR02_0340–SSR02_0307	6308278–5612501	2.8376	4.3066	7.2472
*α*-Toc	2(D1b)	353	SSR02_1774–SSR02_0458	50374310–8328227	2.0848	1.9812	−3.6801
*γ*-Toc	2(D1b)	755	SSR02_0179–SSR02_0146	3040487–2464018	2.1762	10.9363	−10.6354
*α*-Toc	4(C1)	104	Satt338–Satt180	46964891–47299563	3.7889	3.7726	−5.0428
*γ*-Toc	6(C2)	59	Satt643–Sat_213	16057506–14601388	2.2208	5.7178	−7.8969
*α*-Toc	10(O)	141	Satt345–Sat_321	12172070–2471540	3.4919	5.0583	5.8561
*α*-Toc	11(B1)	53	Sat_095–Satt638	29344200–6961206	3.609	4.8946	5.7481
total-Toc	13(F)	214	Satt657–Satt490	38558064–35557764	2.2513	10.5033	−16.3965
*α*-Toc	13(F)	35	Sat_387–Satt252	10787056–5376564	3.0948	4.9516	5.7792
*δ*-Toc	15(E)	184	SSR15_0305–Satt355	6708675–32216247	2.8183	4.2166	−11.5413
*δ*-Toc	18(G)	24	Satt303–Satt472	22150272–58136231	2.9879	17.9714	15.3252
total-Toc	18(G)	75	Satt472–Satt038	58136231–1343760	2.2344	8.2001	14.5236
*α*-Toc	18(G)	96	Satt038–Sat_164	1343760–53656448	6.0305	4.3014	5.3941
*γ*-Toc	18(G)	82	Satt472–Satt038	58136231–1343760	3.2412	6.9263	8.6665
*α*-Toc	19(L)	86	Satt527–Satt664	42835228–46109700	2.9972	4.2565	5.3898
*α*-Toc	20(I)	41	Sat_170–Sct_189	38657685–45550622	2.7995	4.9808	−5.8007
*α*-Toc	20(I)	116	Sct_189–Satt239	45550622–24129710	2.1183	0.5989	−2.9987

**Table 5:** The eQTLs for target genes of *MPBQ*、*TC* and *HPT*.

Trait	eQTL	Gm (LG)	Marker	Marker interval	Position	LOD	PVE (%)
MPBQ	qMPBQD1b_1	2(Dlb)	SSR02_0458	SSR02_0458–SSR02_0520	376	3.02	2.07
	qMPBQE_1	15(E)	Satt355	Satt355–Satt685	193	2.82	6.53
	qMPBQI_1	20(I)	Sct_189	Sct_189–Satt239	103	3.82	7.15
TC	qTCC1_1	4(C1)	Satt338	Satt338–Satt180	103	2.53	3.49
	qTCE_1	15(E)	Satt355	Satt355–Satt685	195	3.46	7.20
	qTCG_1	18(G)	Satt303	Satt303–Satt472	12	3.40	6.71
	qTCG_2	18(G)	Satt472	Satt472–Satt038	82	6.18	2.86
	qTCI_1	20(I)	Sct_189	Sct_189–Satt239	106	4.18	6.52
	qTCI_2	20(I)	Satt239	Satt239–SSR20_1252	124	3.46	5.34

eQTL: The nomenclature of the eQTL included four parts: QTL, trait, linkage group name and QTL order in the linkage group, respectively.

Position: from the left marker of the interval on each linkage group.

### Analysis of the Candidate Genes Regulating Toc Concentration on Chr.02

For association analysis, a total of 177 candidate genes were screened in the 200-kbp flanking region of the identified nineteen QTNs (**Table S4**). Except for the seven genes with an unknown or uncharacterized protein domain, the other 170 candidates were classified and were mainly related to secondary metabolism, lipid metabolism, signaling, cell, protein synthesis/modification/degradation, and RNA regulation of transcription (**Figure S2**). Of these genes, some were directly or indirectly related to the tocopherol metabolism pathways. *Glyma.18G115500* (located near QTN rs14143396 on Chr.18) was derived from the superfamily of NAD(P)-linked oxidoreductase. Two genes belonging to the zinc finger superfamily, including *Glyma.01G021100* and *Glyma.20G113200* located near QTN rs2162876 on Chr.01 and rs35562231 on Chr.20, respectively, might aid in the soybean Toc concentration with higher expressions in soybean seeds and pods (https://soybase.org/) (Park et al., 2019). *Glyma.02G102900* is a protein containing plant U-box domain (PUB26), which triggers the accumulation of Toc content by regulating the transient burst of reactive oxygen species (ROS) (Kadota et al., 2014; Li et al., 2014; Stahl et al., 2019). *Glyma.02G101300* (located near QTN rs9337368 on Chr.02), belonging to cytochrome b561/ferric reductase transmembrane protein family, participates in the regeneration of ascorbic acid; ascorbic acid-glutathione circulation is related to the regeneration of tocopherol phenolic groups, the oxidized form of tocopherol, and the results of the reaction of tocopherol with lipid peroxygen radicals (Kamal-Eldin and Appelqvist, 1996; Lane et al., 2015).

**Figure 5:**
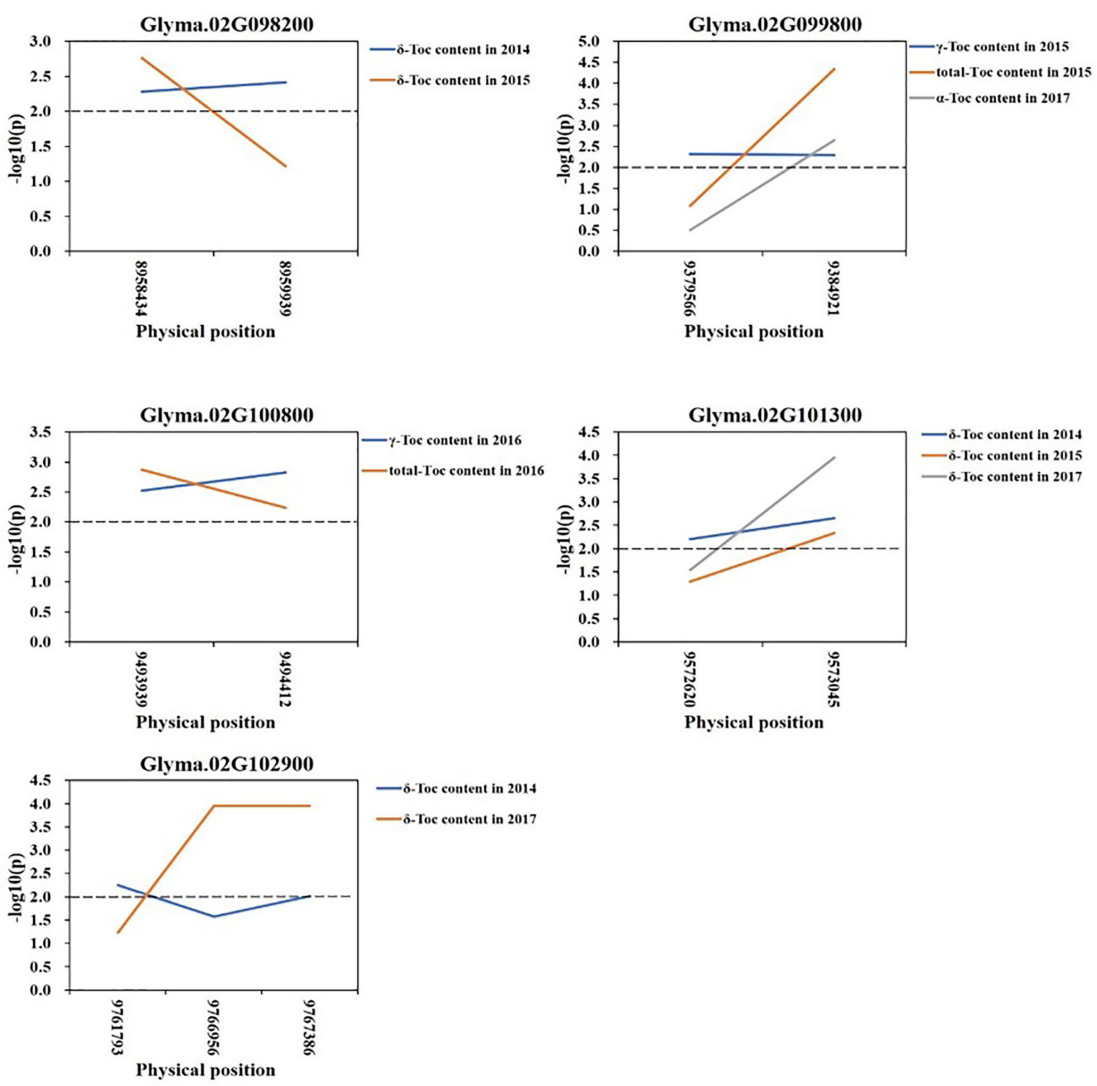
Candidate gene-based association. Gene-based association analysis of candidate genes with SNPs that were significantly correlated to tocopherol content. Horizontal line indicated that the threshold was set to 2.0.

**Figure 6:**
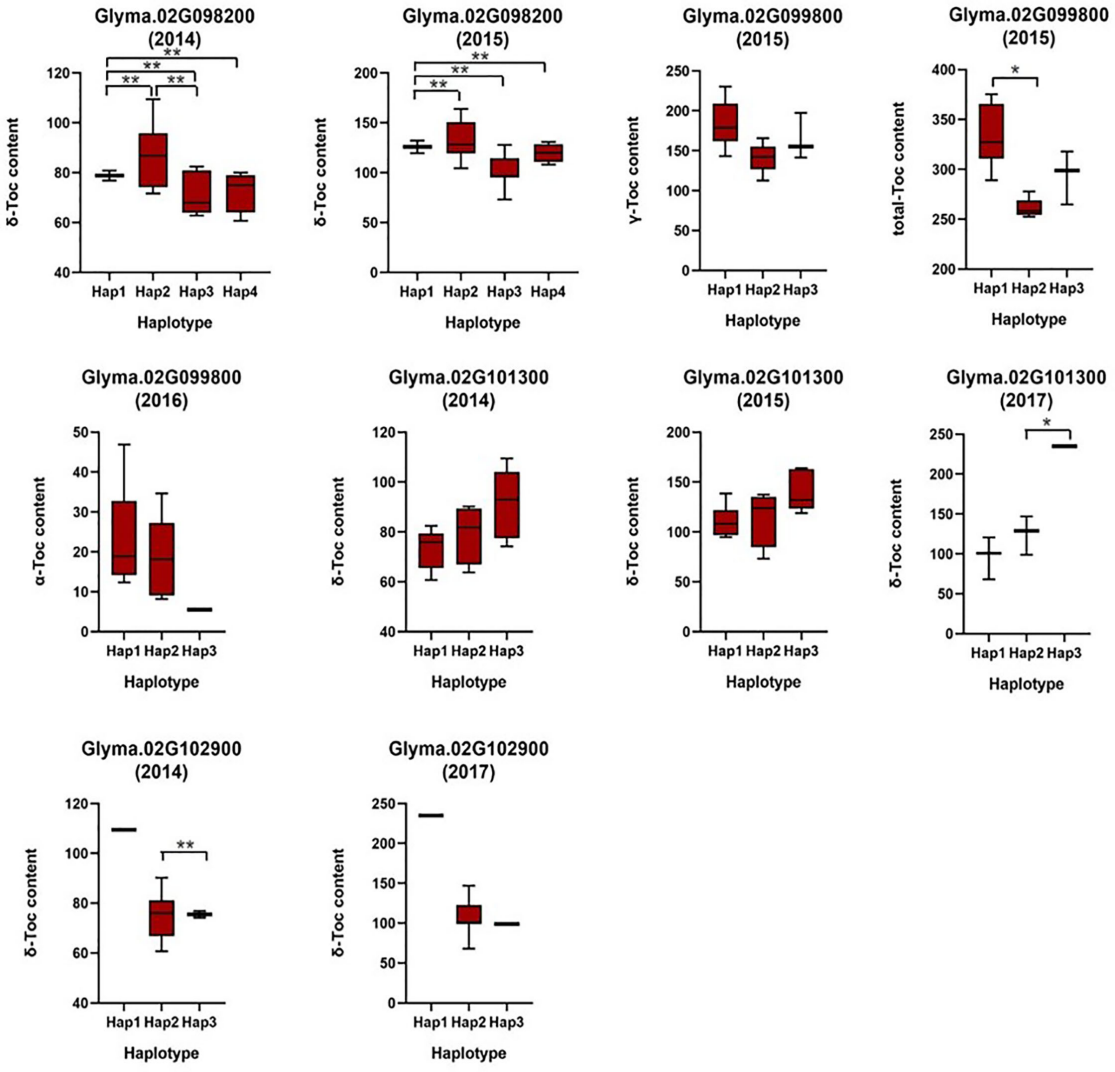
Haplotypes analysis of genes that related to tocopherol content. The * and ** was significance at p < 0.05 and p < 0.01, respectively.

Based on the loci detected by GWAS, QTL, and eQTL analysis, a stable locus on Chr.02 was identified to be highly associated with Toc concentration in soybean seeds. Therefore, a total of 42 genes in the 200-kb flanking region of the QTL (located in the interval of SSR02_0458–SSR02_0520), eQTL (qMPBQD1b_1, located in the interval of SSR02_0458–SSR02_0520), and QTN (rs9337368) were considered as candidate genes (**Table 6**). To verify whether these candidates were associated with individual or total Toc, a candidate gene-based association was conducted. A total of 2,975 SNPs of 42 candidate genes were obtained from the genome re-sequencing of 20 soybean lines (10 higher/lower level of Toc concentration lines). Of them, 108 SNPs from 25 candidate genes were found reaching the threshold with −log_10_(P) ≥ 2. The stable SNPs detected in multiple years were picked out and considered to be significantly associated with Toc concentration; the genes containing these stable SNPs were then referred to the potential ones. Finally, a total of 11 SNPs from five potential genes (*Glyma.02G098200* 2 SNPs; *Glyma.02G099800* 2 SNPs; *Glyma.02G100800* 2 SNPs; *Glyma.02G101300* 2 SNPs; *Glyma.02G102900* 3 SNPs) were identified (**Figure 5** and **Table S5**). For the haplotype analysis, at least two haplotypes existed for each of the five genes, and the individual or total Toc with these different haplotypes exhibited significant or highly significant differences (**Figure 6**).

**Table 6:** Candidate genes in overlapping regions detected by QTL, eQTL, and GWAS.

Gene	Chr.	Start position	Stop position	Distance to left/right QTL (kb)	Distance to eQTL (kb)	Distance to SNP (kb)	Annotation
Glyma.02G098200	Chr02	8959322	8968035	631.10/983.737	631.10	378.05	Transmembrane amino acid transporter family protein
Glyma.02G098300	Chr02	8974511	8999545	646.28/968.548	646.28	362.86	phytochrome and flowering time regulatory protein (PFT1)
Glyma.02G098400	Chr02	9036077	9036352	707.85/906.982	707.85	301.29	Protein with RNI-like/FBD-like domains
Glyma.02G098500	Chr02	9038015	9060145	709.79/905.044	709.79	299.35	phytochrome and flowering time regulatory protein (PFT1)
Glyma.02G098600	Chr02	9101997	9106449	773.77/841.062	773.77	235.37	Endosomal targeting BRO1-like domain-containing protein
Glyma.02G098700	Chr02	9141033	9149017	812.81/802.026	812.81	196.34	Metallo-hydrolase/oxidoreductase superfamily protein
Glyma.02G098800	Chr02	9169020	9171899	840.79/774.039	840.79	168.35	myb-like transcription factor family protein
Glyma.02G099000	Chr02	9195657	9195881	867.43/747.402	867.43	141.71	ROTUNDIFOLIA like 21
Glyma.02G099100	Chr02	9220377	9222397	892.15/722.682	892.15	116.99	C2H2-like zinc finger protein
Glyma.02G099200	Chr02	9257064	9258661	928.84/685.995	928.84	80.30	allene oxide cyclase 4
Glyma.02G099300	Chr02	9259923	9271860	931.70/683.136	931.70	77.45	methionine aminopeptidase 1B
Glyma.02G099400	Chr02	9293211	9293733	964.98/649.848	964.98	44.16	glutathione S-transferase tau 7
Glyma.02G099500	Chr02	9306726	9308948	978.50/636.333	978.50	30.64	AP2/B3 transcription factor family protein
Glyma.02G099800	Chr02	9374053	9384823	1045.83/569.006	1045.83	36.69	Di-glucose binding protein with Leucine-rich repeat domain
Glyma.02G099900	Chr02	9401424	9403579	1073.20/541.635	1073.20	64.06	galacturonosyltransferase-like 3
Glyma.02G100000	Chr02	9418107	9423906	1089.88/524.952	1089.88	80.74	STT7 homolog STN7
Glyma.02G100100	Chr02	9429539	9430299	1101.31/513.52	1101.31	92.17	ROTUNDIFOLIA like 15
Glyma.02G100200	Chr02	9440858	9446878	1112.63/502.201	1112.63	103.49	NAC (No Apical Meristem) domain transcriptional regulator superfamily protein
Glyma.02G100300	Chr02	9449378	9454175	1121.15/493.681	1121.15	112.01	Protein kinase superfamily protein
Glyma.02G100400	Chr02	9454764	9457069	1126.54/488.295	1126.54	117.40	Protein kinase superfamily protein
Glyma.02G100500	Chr02	9458910	9462064	1130.68/484.149	1130.68	121.54	Leucine-rich repeat protein kinase family protein
Glyma.02G100800	Chr02	9493864	9495581	1165.64/449.195	1165.64	156.50	Eukaryotic aspartyl protease family protein
Glyma.02G101300	Chr02	9565694	9576957	1237.47/377.365	1237.47	228.33	Cytochrome b561/ferric reductase transmembrane protein family
Glyma.02G101400	Chr02	9596487	9600839	1268.26/346.572	1268.26	259.12	2-oxoglutarate (2OG) and Fe(II)-dependent oxygenase superfamily protein
Glyma.02G101500	Chr02	9603161	9609480	1274.93/339.898	1274.93	265.79	decapping 5
Glyma.02G101700	Chr02	9635465	9642262	1307.24/307.594	1307.24	298.10	decapping 5
Glyma.02G101800	Chr02	9646459	9655022	1318.23/296.6	1318.23	309.09	nuclear matrix constituent protein-related
Glyma.02G101900	Chr02	9656994	9660818	1328.77/286.065	1328.77	319.63	Leucine-rich repeat (LRR) family protein
Glyma.02G102000	Chr02	9666417	9673757	1338.19/276.642	1338.19	329.05	Tetratricopeptide repeat (TPR)-like superfamily protein
Glyma.02G102400	Chr02	9687071	9689680	1358.84/255.988	1358.84	349.70	Thioredoxin superfamily protein
Glyma.02G102500	Chr02	9706607	9709983	1378.38/236.452	1378.38	369.24	Putative endonuclease or glycosyl hydrolase
Glyma.02G102600	Chr02	9710565	9715971	1382.34/232.494	1382.34	373.20	Protein kinase superfamily protein
Glyma.02G102700	Chr02	9722998	9730991	1394.77/220.061	1394.77	385.63	PLC-like phosphodiesterases superfamily protein
Glyma.02G102800	Chr02	9733111	9737918	1404.88/209.948	1404.88	395.74	PLC-like phosphodiesterases superfamily protein
Glyma.02G102900	Chr02	9762548	9766635	1434.32/180.511	1434.32	425.18	plant U-box 26
Glyma.02G103200	Chr02	9795987	9799196	1467.76/147.072	1467.76	458.62	basic helix-loop-helix (bHLH) DNA-binding superfamily protein
Glyma.02G103300	Chr02	9805442	9814109	1477.22/137.617	1477.22	468.07	RING/U-box superfamily protein
Glyma.02G103400	Chr02	9820787	9821647	1492.56/122.272	1492.56	483.42	zinc ion binding; nucleic acid binding
Glyma.02G103500	Chr02	9836681	9838714	1508.45/106.378	1508.45	499.31	S-adenosyl-L-methionine-dependent methyltransferases superfamily protein
Glyma.02G103600	Chr02	9841746	9843168	1513.52/101.313	1513.52	504.38	Ribosomal protein S5 domain 2-like superfamily protein
Glyma.02G103700	Chr02	9844516	9849873	1516.29/98.543	1516.29	507.15	Plant protein of unknown function (DUF869)
Glyma.02G103800	Chr02	9854347	9861255	1526.12/88.712	1526.12	516.98	RING/U-box superfamily protein

**Figure 7:**
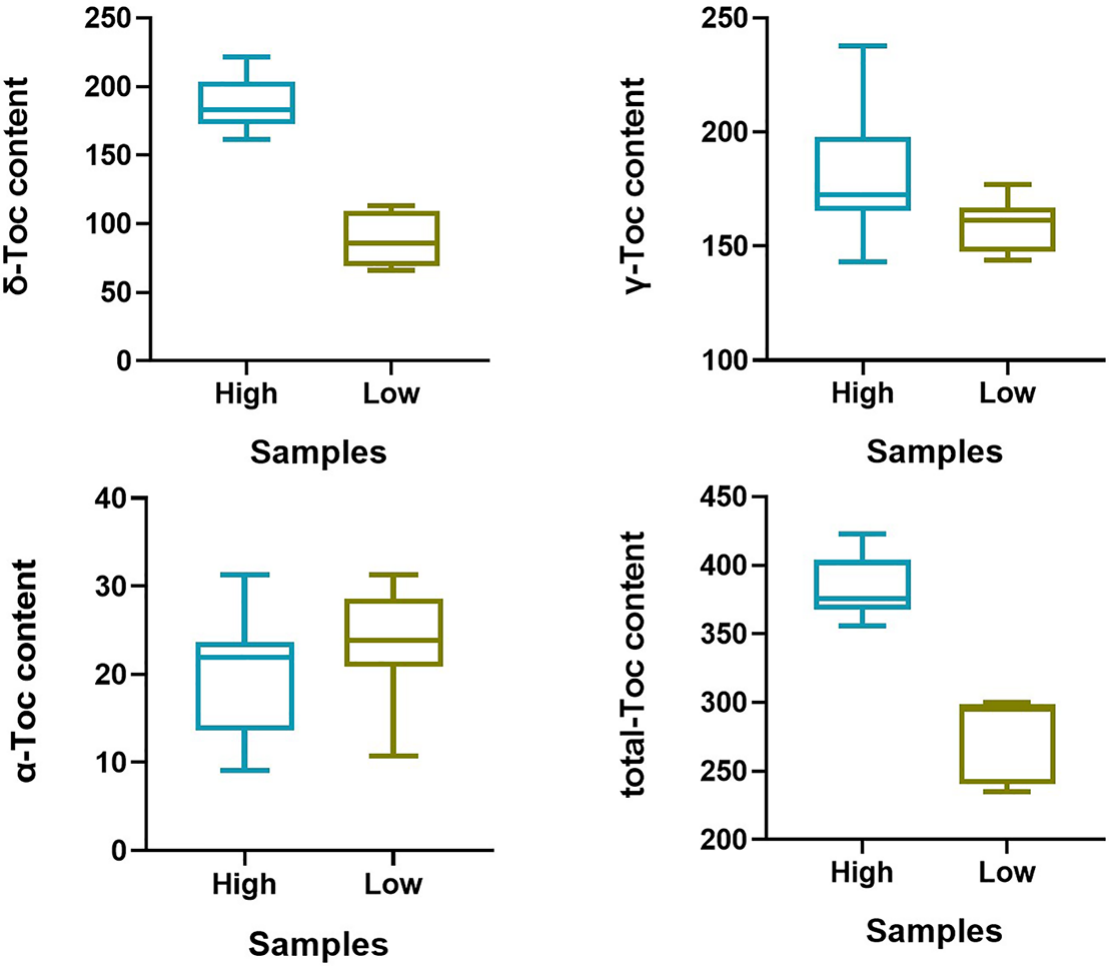
Tocopherol content of 16 germplasms at R7 growth period. “High” represent the higher accessions (Line 1–Line 8), “Low” represent the lower accessions (Line 9–Line 16).

**Figure 8:**
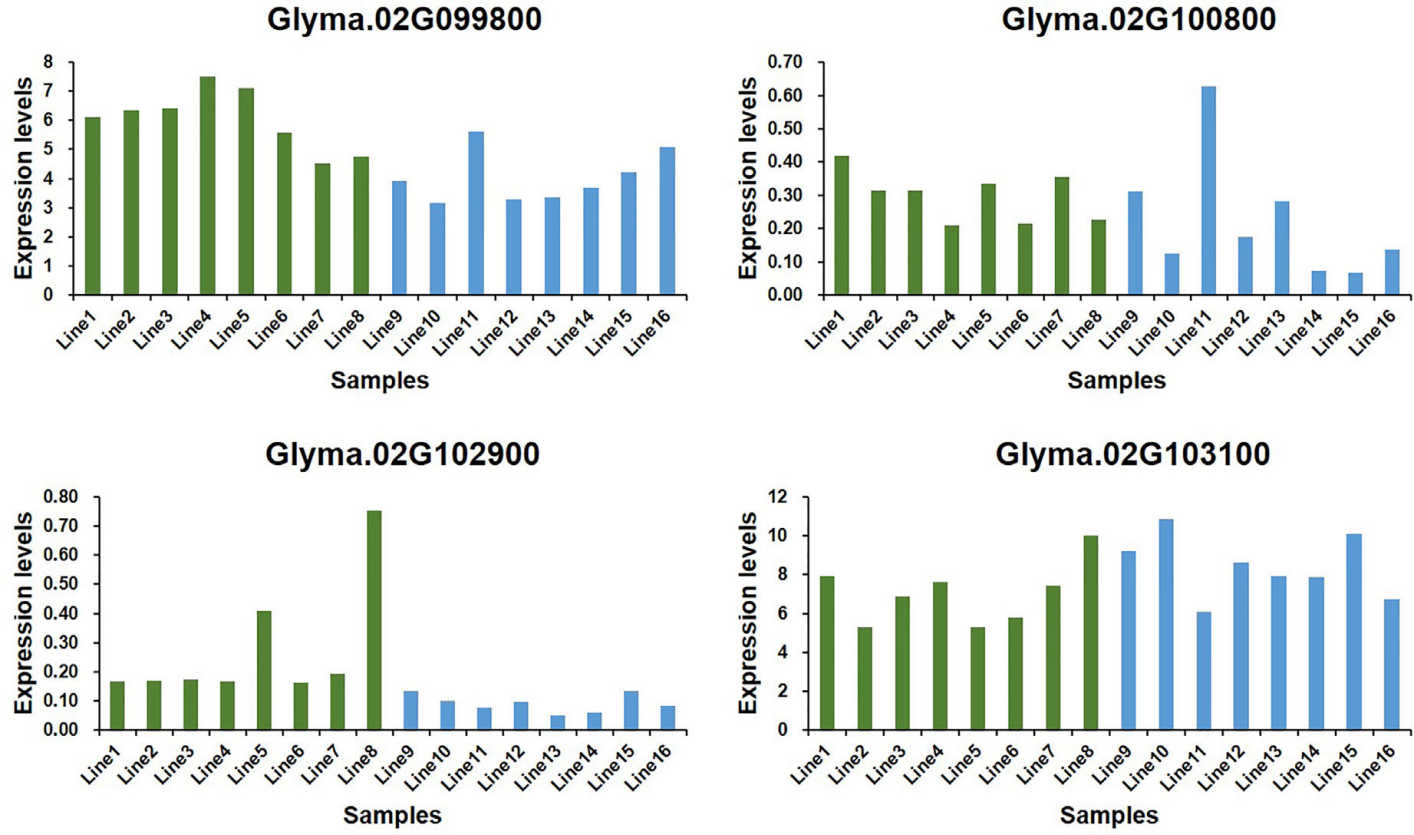
Expression levels of candidate genes in 16 soybean germplasms at R7 growth period.

To predict the potential role of candidate genes in the regulation of Toc content, pods from 16 characteristic soybean germplasms at development stages (R7) were selected to determine the content of *δ*-Toc, *γ*-Toc, *α*-Toc and total-Toc (**Figure 7**), and to analyze the expression levels of five potential genes. Of them, only *Glyma.02g098200* was not expressed in immature soybean seeds, and the expression levels of the other four genes varied greatly among soybean germplasms (**Figure 8**). A statistical analysis for the expression levels of these five genes were conducted. The result showed that the expression levels of the five potential genes in 16 germplasms varied over a wide range and the expression levels among the lines with higher and lower tocopherol contents were different, including *Glyma.02G099800* (Line 1 to Line 8 were from 4.53 to 7.52, Line 9 to Line 16 were from 3.16 to 5.63), *Glyma.02G100800* (Line 1 to Line 8 were from 0.21 to 0.42, Line 9 to Line 16 were from 0.07 to 0.63), *Glyma.02G102900* (Line 1 to Line 8 were from 0.17 to 0.75, Line 9 to Line 16 were from 0.05 to 0.13), *Glyma.02G101300* (Line 1 to Line 8 were from 5.27 to 9.99, Line 9 to Line 16 were from 6.05 to 10.86). Among them, the range of the expression levels of *Glyma.02G099800* and *Glyma.02G102900* in higher Toc lines (Line 1 to Line 8) was much higher than those of lower Toc lines (Line 9 to Line 16) (**Table S6**). An analysis of Pearson’s correlation coefficient was performed to analyze the correlations between expression levels and tocopherol contents of the five potential genes. The results showed that the expression levels of *Glyma.02G099800*, *Glyma.02G100800* and *Glyma.02G102900* in pods were significantly correlated with total Toc content or its components, and *Glyma.02G101300* showed no significant correlation with Toc content (**Table S7**). Among them, the expression level of *Glyma.02G099800* showed significantly positive correlation (P < 0.05) with the *γ*-Toc content and total Toc content in soybean seeds among tested germplasms. The expression level of *Glyma.02G100800* showed significantly positive correlation (P < 0.05) with the content of *δ*-Toc and showed the opposite correlation with *α*-Toc content. In addition, the expression level of *Glyma.02G102900* was significantly positively correlated with *δ*-Toc content (P < 0.01) and total-Toc content (P < 0.05) (**Table S7**). These genes with beneficial haplotypes would be of great value in regulating individual and total Toc concentration of soybean seeds.

## Discussion

Toc compounds, belonging to the vitamin E family, are lipophilic antioxidants that are beneficial to human health because they can prevent the oxidation of unsaturated fatty acids (Shaw et al., 2016). Compared to other oil crops, soybean oil contains a higher total Toc concentration with the dominant form being *γ*-Toc, which comprises 70% of the total Toc content. The vitamin E activity of *γ*-Toc is only moderate due to its lower affinity with the hepatic tocopherol transfer protein. The *α*-Toc, with a lower content in oil, has the highest activity (Bramley et al., 2000). Therefore, increasing the *α*-Toc and total Toc content in soybean seeds is important. In this four-year study, a germplasm collection of 180 soybean accessions in Harbin was used for measuring individual and total Toc content. Toc levels had a wide range of variation among the different accessions but a relatively smaller range of variation among the different years. The test samples containing a higher level of *α*-Toc and total Toc might be the useful sources for the breeding of new soybean cultivars.

Nearly thirty QTLs have been identified associated with individual or total Toc *via* different mapping populations, and most of these are specific to the genetic background (www.soybase.org). Li et al. (2010) detected four, eight, four, and five QTLs related to *α*-Toc, *γ*-Toc, *δ*-Toc, and total Toc from “OAC Bayfield,” respectively. Shaw et al. (2017) identified 26 SSR markers linked to QTL with individual and total Toc content from the cross of ‘OAC Bayfield’ and ‘OAC Shire.’ In the present study, 18 QTLs mapped to 10 chromosomes and nine eQTLs on five different chromosomes were associated with individual and total Toc content through linkage mapping. Additionally, a GWAS for Toc-related was also performed, and 19 stable QTNs detected in more than two years were identified. Among these QTNs, 16 loci were regarded as novel for soybean Toc, and the other three contained similar genomic regions known as QTLs for Toc concentration reported by Shaw et al. (2017). The rs37703714 on Chr.17 and rs33069111 and rs35562231 on Chr.20 were close to “Seed tocopherol, gamma 3-6” and “Seed tocopherol, total 3-3,” respectively, which were detected between the SSR markers of “Sat_354–Satt135” and “Satt270–BARC-041717-08071” using the “OAC Bayfield × OAC Shire” mapping population. The allele effects in these identified QTNs were evaluated, and the results showed that these alleles occurred on diverse soybean accessions. The soybean accessions with the “beneficial” allele in these identified QTNs had a higher individual or total Toc concentration than accessions with the “inferior” allele. Thus, these QTNs, exhibiting more potential in manipulating individual or total Toc, might be useful for MAS.

Few definite genes associated with individual or total Toc have been characterized or cloned, except for the genes encoding the key enzymes involved in Toc metabolism. For accurately screening candidate genes, a total of 42 genes in the 200-kb flanking region of the locus on Chr.02 co-detected by QTL, eQTL, and GWAS analysis were selected and classified into different functional groups using the Gene Ontology database (http://geneontology.org/). Using a gene-based association by the GLM method, five genes (*Glyma.02G098200*, *Glyma.02G099800*, *Glyma.02G100800*, *Glyma.02G101300*, and *Glyma.02G102900*) were determined to be significantly related to *α*-Toc, *γ*-Toc, *δ*-Toc and total Toc in soybean seeds. Further expression level analysis of these five genes showed that the expression levels of *Glyma.02G099800* and *Glyma.02G102900* were significantly correlated with Toc content in soybean seeds, which might regulate Toc content. *Glyma.02G102900* is a plant U-box domain-containing protein (PUB26), as an E3 ligase, which targets BIK1 for degradation and negatively regulates BIK1-mediated immunity. BIK1 is of central importance to the plant immune system, and it regulates transient bursts of reactive oxygen species (ROS) (Kadota et al., 2014; Li et al., 2014). ROS has a triggering function in the pathogen-inducible biosynthesis of tocopherols; the elevated ROS can trigger the accumulation of Toc content (Stahl et al., 2019). *Glyma.02G099800*, belonging to Di-glucose binding protein with Leucine-rich repeat domain, is a Receptor-like kinase (RLK). RLKs mediate a large amount of signal transmission information on the cell surface and acts as a crucial regulator in the development process (Afzal et al., 2008; Gish and Clark, 2011). The role of this gene in Toc metabolism needs to be further explored. Additional studies on the functions and mechanisms of these candidate genes are planned.

## Data Availability Statement

All datasets generated for this study are included in the article/**Supplementary Material**.

## Author Contributions

MS, YJ, and HL conceived the study and contributed to population development. YZ and HZ contributed to genotyping. JL and WT contributed to phenotypic evaluation. LQ, WL, XZ, and YH contributed to experimental design and writing the paper. All authors contributed to the article and approved the submitted version.

## Funding

This study was financially supported by the Heilongjiang Provincial Project (GJ2018GJ0098, JC2018007, GX17B002, C2018016), the National Key R & D Project (2017YFD0101306, 2016YFD0100304, 2017YFD0101302), the Chinese National Natural Science Foundation (31701446,31671717, 31471517, 31971967, 31871650), the National Project (2014BAD22B01, 2016ZX08004001-007), the Youth Leading Talent Project of the Ministry of Science and Technology in China (2015RA228), The National Ten-thousand Talents Program, Postdoctoral Fund in Heilongjiang Province (LBH-Z15017, LBH-Q17015), The national project (CARS-04-PS04), The ‘Youth Innovation Talent’ Project of the general undergraduate universities in Heilongjiang province (UNPYSCT-2016145), the ‘Academic Backbone’ Project of Northeast Agricultural University (17XG22), Student Innovation Practical Training (SIPT). The funding bodies had no role in study design, data collection and analysis, decision to publish, or preparation of the manuscript.

## Conflict of Interest

HZ was employed by the company Biomarker Technologies Corporation.

The remaining authors declare that the research was conducted in the absence of any commercial or financial relationships that could be construed as a potential conflict of interest.

## Supplementary Material

Supplementary Figure S1 Manhattan plot of association mapping of the tocopherol content in soybean.

Supplementary Figure S2 Functional classifications of the candidate genes of tocopherol content in soybean.

Supplementary Table S1 The information of 180 soybean accessions.

Supplementary Table S2 PCR primers used for expression analysis by RT-qPCR.

Supplementary Table S3 Information of SSR markers among RILs.

Supplementary Table S4 Gene models in the flanking regions of peak SNP by GWAS.

Supplementary Table S5 Haplotype analysis of candidate genes.

Supplementary Table S6 Basic genetic parameter statistics for expression levels of genes.

Supplementary Table S7 Correlation analysis of tocopherol contents with expression levels of genes.
