# Supplementary figures and images for: Identification of Loci and Candidate Genes Analyses for Tocopherol Concentration of Soybean Seed
Source: Front Plant Sci. 2020 Sep 9;11:539460. doi: 10.3389/fpls.2020.539460 (PMC7509058; doi:10.3389/fpls.2020.539460)

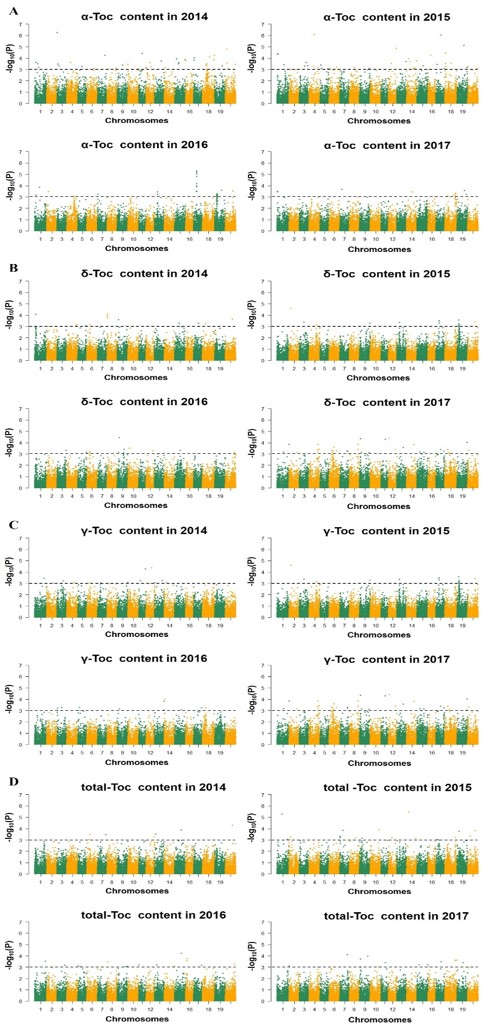

Supplement: Supplementary Figure S1 — Manhattan plot of association mapping of the tocopherol content in soybean. [file Image_1.jpeg]

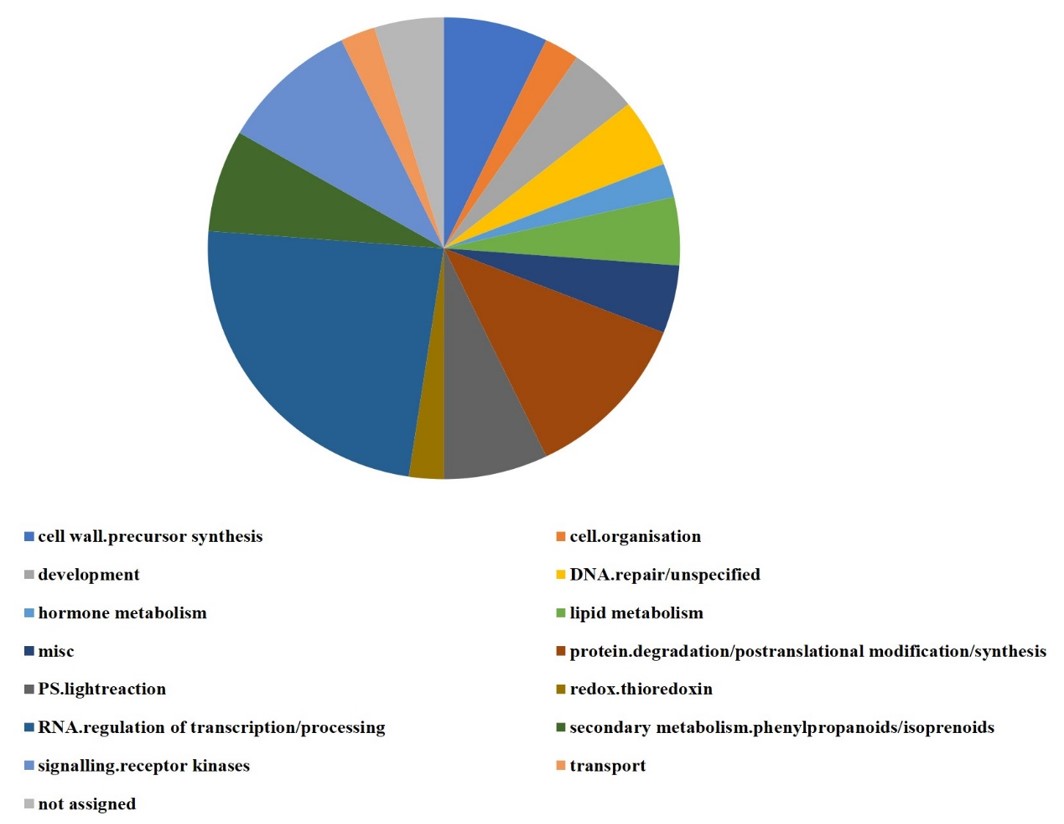

Supplement: Supplementary Figure S2 — Functional classifications of the candidate genes of tocopherol content in soybean. [file Image_2.jpeg]
